# Esquistossomose e o Coração - Em Nome das Doenças Tropicais Negligenciadas e Outras Doenças Infecciosas que Afetam o Coração (Projeto NET-Heart)

**DOI:** 10.36660/abc.20201384

**Published:** 2022-01-11

**Authors:** Edith Liliana Posada-Martínez, Luis Gerardo Gonzalez-Barrera, Kiera Liblik, Juan Esteban Gomez-Mesa, Clara Saldarriaga, Juan Maria Farina, Josefina Parodi, Zier Zhou, Manuel Martinez-Selles, Adrian Baranchuk

**Affiliations:** 1 Ignacio Chavez National Institute for Cardiology Juan Badiano México Ignacio Chavez National Institute for Cardiology – Echocardiography, Juan Badiano – México; 2 Medical Society National Medical Center Mexico City México Medical Society of 20 of November National Medical Center – Cardiology, Mexico City – México; 3 Queen’s University Kingston Ontario Canadá Queen’s University, Kingston, Ontario – Canadá; 4 Valle del Lili Foundation Internal Medicine Department Valle del Cauca Colômbia Valle del Lili Foundation Internal Medicine Department, Valle del Cauca – Colômbia; 5 Cardiovascular Clinic Santa Maria Medellin Colômbia Cardiovascular Clinic Santa Maria - Cardiology and Heart Failure, Medellin – Colômbia; 6 University of Antioquia Medellin Colômbia University of Antioquia, Medellin – Colômbia; 7 Clinica Olivos Buenos Aires Argentina Clinica Olivos – Cardiology, Buenos Aires – Argentina; 8 Cardiovascular Institute of Buenos Aires Buenos Aires Argentina Cardiovascular Institute of Buenos Aires, Buenos Aires – Argentina; 9 Gregorio Maranon General University Hospital Cardiology Service Madrid Espanha Gregorio Maranon General University Hospital Cardiology Service, Madrid – Espanha

**Keywords:** Esquistossomose, Doenças Cardiovasculares, Medicina Tropical

## Abstract

**Fundamento:**

A esquistossomose é uma doença tropical negligenciada que pode levar a complicações cardiovasculares. No entanto, o envolvimento cardiovascular na esquistossomose ainda precisa ser totalmente elucidado, devido ao número limitado de casos e ausência de evidência confiável, uma vez que a doença ocorre tipicamente em locais sem infraestrutura adequada para uma coleta de dados robusta.

**Objetivo:**

Esta revisão sistemática teve como objetivo avaliar as implicações cardiovasculares da esquistossomose, incluindo no diagnóstico e tratamento, e propor um algoritmo para rastrear as manifestações cardiovasculares.

**Métodos:**

Foi realizada uma revisão sistemática nos bancos de dados MEDLINE/PubMed e LILACS, com busca por artigos sobre o comprometimento cardiovascular na esquistossomose.

**Resultados:**

Trinta e três artigos foram considerados para esta revisão: seis artigos de revisão, uma revisão sistemática, um ensaio clínico, 14 estudos observacionais, sete relatos de casos, e quatro séries de casos. O comprometimento cardiovascular inclui um amplo espectro de condições clínicas, tais como isquemia do miocárdio, disfunção ventricular, miocardite, hipertensão arterial pulmonar, e pericardite.

**Conclusões:**

As complicações cardíacas da esquistossomose podem causar incapacidade em longo prazo e morte. O monitoramento clínico, exame físico, eletrocardiograma precoce, e ecocardiograma devem ser considerados como medidas chave para detectar o envolvimento cardiovascular. Dada a ausência de um tratamento eficaz das complicações, são necessários saneamento e educação nas áreas endêmicas para a eliminação desse problema de saúde mundial.

## Introdução

A esquistossomose é uma doença tropical negligenciada (DTN) causada por platelmintos, que são trematódeos do gênero Schistosoma. A doença é endêmica de regiões rurais com má infraestrutura de saúde, e acesso limitado à água potável ou métodos de saneamento da água. A esquistossomose foi incluída no plano de combate às DTNs da Organização Mundial da Saúde (OMS) de 2008-2015.^1^

Segundo a OMS, aproximadamente 240 milhões de pessoas são afetadas pela esquistossomose no mundo, com mais de 90% dos casos registrados na África.^1^ A esquistossomose é transmitida pela penetração de larvas de Schistosoma (cercárias), presentes em água doce, na pele. Uma vez que as larvas penetram na pele, elas invadem o sistema venoso e se espalham por órgãos tais como coração, pulmões, fígado e intestinos. O *Schistosoma mansoni* é a principal espécie que infecta os humanos, e pode levar a eventos cardiovasculares fatais. Em relatos de casos publicados, miocardite, pericardite, e isquemia do miocárdio foram documentados na fase aguda da doença.^2,3^ Esses desfechos cardiovasculares são pouco compreendidos, devido ao número limitado de casos e ausência de registros.^4^ Pacientes com complicações cardiovasculares agudas podem ainda se apresentarem assintomáticos, contribuindo para deficiências na coleta de dados. A complicação mais importante da esquistossomose é a hipertensão arterial pulmonar (HAP).^5-7^ Sinais e sintomas de pacientes com HAP associada à esquistossomose não são diferentes daqueles causados por HAP com outras etiologias.

Estima-se que a esquistossomose seja a principal causa de HAP em países endêmicos. Apesar disso, o diagnóstico é limitado a regiões com acesso a equipamentos médicos adequados e, atualmente, não existem medicamentos específicos para a HAP associada à esquistossomose. O agente farmacológico mais utilizado é o praziquantel (PZQ), que presumivelmente previne a progressão da doença por reversão do remodelamento vascular.

Esta revisão sistemática é parte do projeto “NET-Heart” (*Neglected Tropical Diseases and Other Infectious Diseases Affecting the Heart*, ou Doenças tropicais Negligenciadas e Outras Doenças Infecciosas que Afetam o Coração), uma iniciativa da seção “Líderes Emergentes” da Sociedade Interamericana de Cardiologia (SIAC).^8-10^ O objetivo deste estudo foi expandir o conhecimento do impacto da DTN sobre a saúde cardiovascular. O objetivo desta revisão foi apresentar uma visão geral do envolvimento cardiovascular na esquistossomose e propor um algoritmo para o diagnóstico.

## Métodos

**Figura 1:**
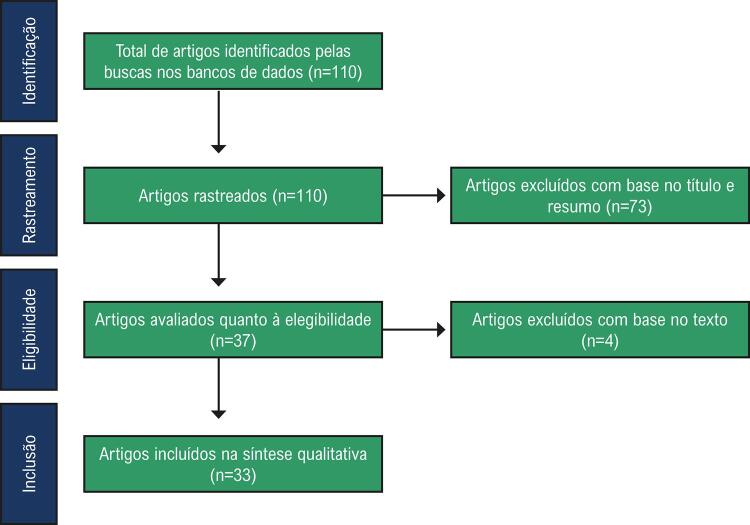
– *Fluxograma da metodologia PRISMA.*

Uma revisão sistemática da literatura foi conduzida seguindo-se o delineamento do projeto NET-Heart.^8,11^Foi realizada uma busca nos bancos de dados MEDLINE/PubMed e LILACS por qualquer associação entre esquistossomose e envolvimento cardiovascular, sem restrição de data. Foram analisados somente estudos em inglês, envolvendo humanos. Artigos sem o texto completo disponível foram excluídos. As palavras chaves utilizadas, de acordo com a terminologia MESH foram: “esquistossomose, “coração”, “cardíaco”, “pericárdio”, “pericardite”, e “doença cardiovascular”. Os artigos foram rastreados por dois investigadores independentes (ELPM e LGGB). A concordância entre observadores, calculada pelo coeficiente Kappa, foi de 0,93. Discrepâncias foram resolvidas por consenso. Uma busca manual foi também realizada a partir das referências dos artigos selecionados. A busca resultou em 110 artigos, dos quais 33 artigos foram incluídos nesta revisão sistemática: seis artigos de revisão, uma revisão sistemática, um ensaio clínico, 14 estudos observacionais, sete relatos de casos, e quatro séries de casos (Figura 1). A Tabela 1 (material suplementar) resume os estudos considerados nesta revisão.

**Tabela 1:** – Sintomas e sinais de hipertensão arterial pulmonar associada à esquistossomose

Sintomas	Sinais	
Hipertensão arterial pulmonar		
Dispneia	Disfonia	
Fraqueza	Hemoptise	
Angina	Impulso paraesternal esquerdo	
Síncope	Componente pulmonar acentuado do segundo som cardíaco	
Tosse	Terceira bulha cardíaca no ventrículo esquerdo	
Náusea e vômito	Sopro sistólico paraesternal da regurgitação tricúspide	
	Sopro diastólico da regurgitação pulmonar	
**Insuficiência cardíaca direita**		
Dispneia		Pletora jugular
Dor abdominal		Ascite
Edema de membros		Hepatomegalia
Fadiga		Edema periférico

*Dados adaptados de Galie et al. 2015 ESC/ERS Guidelines for the diagnosis and treatment of pulmonary hypertension: The Joint Task Force for the Diagnosis and Treatment of Pulmonary Hypertension of the European Society of Cardiology (ESC) and the European Respiratory Society (ERS). Eur Heart J. 2016;37(1):67-119.*

## Resultados

### Epidemiologia

A esquistossomose é uma doença parasitária crônica causada por platelmintos do gênero Schistosoma. Entre os indivíduos infectados, aproximadamente 120 milhões de pessoas são sintomáticas, e 20 milhões apresentam formas graves da doença, incluindo a forma hepatoesplênica e a forma urinária.^12^

**Figura 2:**
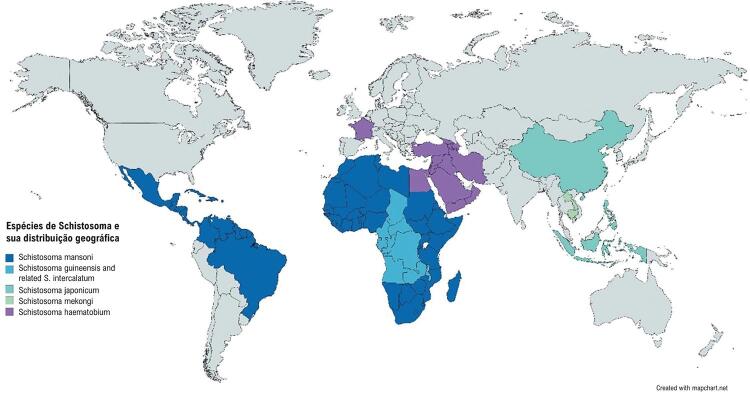
– *Espécies de Schistosoma e sua distribuição geográfica; imagem adaptada da Organização Mundial da Saúde.^1^*

A esquistossomose é considerada endêmica na América do Sul, Caribe, sudeste asiático, e África (Figura 2). A África é a área mais afetada, com mais de 90% das 41 mortes e 1,7 milhões de anos de vida ajustados por incapacidade atribuídos a essa doença anualmente. Ainda, a expansão do turismo internacional a países endêmicos tem resultado em um aumento crescente de infecções entre os trabalhadores. Em termos de envolvimento cardiovascular, a esquistossomose é uma das principais causas de HAP em todo o mundo, contribuindo para 30,8% de todos os casos de HAP nas áreas endêmicas.^13^

### Patofisiologia e envolvimento cardiovascular

**Figura 3:**
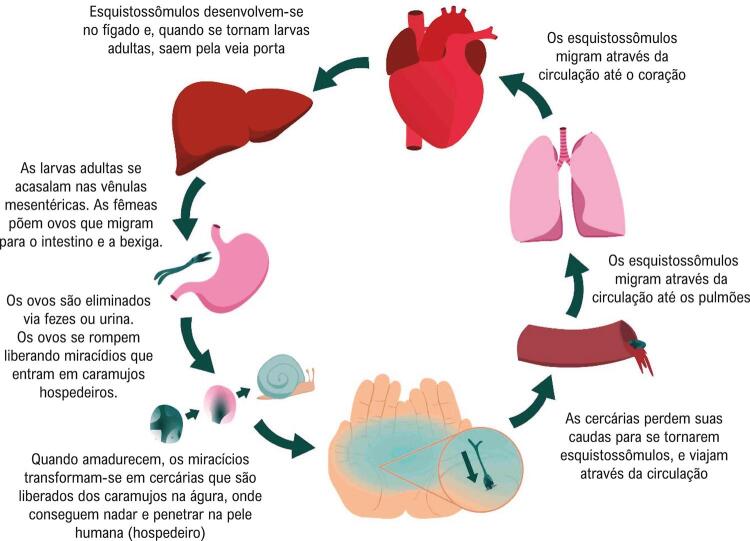
– *Ciclo de vida do Schistosoma.*

Existem cinco principais espécies de Schistosoma que infectam os humanos. *S. mansoni, S. haematobium*, e *S. japonicum* causam a maioria das infecções no homem.^13^ O ciclo de vida do Schistosoma está apresentado na Figura 3.

A HAP causada pela esquistossomose está particularmente associada com a forma hepatoesplênica da infecção pelo *S. mansoni*.^14^ Os ovos de Schistosoma podem passar para o fígado através dos vasos colaterais portossistêmicos e se depositarem nos pulmões. Os ovos induzem resposta imune predominantemente de células T *helper* do tipo 2, resultando na formação de granuloma. Foi demonstrado que as interleucinas (IL-3 e IL-4) estimulam a liberação do fator de transformação de crescimento beta, levando à remodelação e lesões angiomatosas e plexiformes.^13^ Ainda, espécies cujos ovos encontram-se no plexo venoso vesical podem alcançar diretamente os pulmões.^15-17^

A fisiopatologia da HAP associada à esquistossomose pode ser resumida a seguir: obstrução mecânica da circulação pulmonar pelos ovos; inflamação que leva à disfunção das células endoteliais; e hipertensão portal devido à fibrose periportal, levando à sobrecarga pulmonar e consequente disfunção das células endoteliais.^5^

A esquistossomose aguda, conhecida como febre de Katayama, pode ter efeitos cardiovasculares, tais como miocardite, isquemia do miocárdio assintomática, e pericardite.^2,18^As espécies com envolvimento cardíaco na fase aguda são *S. haematobium, S. mansoni,* e *S. japonjicum*. A miocardite e a pericardite durante a esquistossomose aguda podem estar relacionadas a uma resposta alérgica induzida pelo Schistosoma, em que os eosinófilos têm um papel essencial.^19-21^ O mecanismo da isquemia do miocárdio como uma consequência da esquistossomose não foi descrito, e é raramente relatado;^2,3^ pode ocorrer como consequência da compressão da artéria coronária esquerda causada pela dilatação da artéria pulmonar. Dilatação grave da artéria pulmonar pode resultar em ruptura, causando tamponamento cardíaco.^17^

### Sintomas

A HAP induzida por esquistossomose é uma condição que pode ser assintomática. No entanto, nos estágios posteriores da doença, os pacientes podem apresentar sintomas de insuficiência cardíaca direita, tais como dispneia, edema bilateral de membros inferiores, e taquicardia. Sinais e sintomas de HAP na esquistossomose estão descritos na Tabela 1.

Os sintomas são inespecíficos, e principalmente associados à disfunção ventricular direita. No início dos sintomas, os pacientes podem relatar que os sintomas foram induzidos por exercício. Na progressão da doença, os pacientes podem desenvolver insuficiência cardíaca direita avançada, com sintomas de congestão venosa sistêmica.^17,22^ Disfonia pode ser notada, a qual é causada por compressão do nervo laríngeo recorrente. Angina foi relatada em casos que evoluíram para isquemia do miocárdio.^17^ Outros sinais clínicos de HAP associada à esquistossomose incluem hepatomegalia, ascite, edema periférico, e pressão venosa jugular elevada.^17^

Na esquistossomose aguda que envolve o coração, os pacientes com miocardite podem exibir dor torácica. Esses pacientes podem ser assintomáticos, com o diagnóstico feito com base em testes laboratoriais. Ainda, há um relato de um paciente que apresentou perfusão miocárdica tardia do septo, com realce do realce subendocárdico dois meses após a fase aguda, sem sinais clínicos de isquemia.^2^

### Testes diagnósticos

O diagnóstico de esquistossomose requer uma anamnese precisa e orientada, exames físicos, testes laboratoriais, e estudos de imagens. Em áreas endêmicas, deve-se suspeitar de esquistossomose em pacientes com manifestações de HAP. Em países não endêmicos, na presença de sintomas cardiovasculares, uma viagem recente a áreas endêmicas deve ser considerada.

A identificação do parasita é uma parte importante do diagnóstico. No entanto, o exame microscópico de ovos na urina (*S. haematobium*) ou nas fezes (*S. japonicum, S. mansoni*) nem sempre é possível se o parasita se encontra no período pré-patente.^23^ Ainda, os testes sorológicos disponíveis são limitados, uma vez que não discriminam ente infecção ativa e exposição prévia.^12,24^ Finalmente, testes baseados em PCR foram desenvolvidos para a detecção de DNA de Schistosoma nas fezes, soro, e plasma durante todas as fases da doença.^25^

Em relação ao envolvimento cardíaco, pacientes com HAP podem apresentar aumento do átrio direito, hipertrofia ventricular direita, e bloqueio do ramo direito no eletrocardiograma.^19,26^ Além disso, o exame de raio-X mostra típicas artérias pulmonares direita e esquerda proeminentes nesses pacientes.^27^

Na esquistossomose aguda, o ecocardiograma ajuda a identificar miocardite, pericardite, ou isquemia miocárdica.^2,28,29^ Em pacientes com HAP, o exame pode revelar aumento do ventrículo direito, com desvio do septo interventricular para a esquerda, regurgitação tricúspide, hipertrofia da parede livre do ventrículo esquerdo, e aumento na pressão ventricular direita. Ainda, o ecocardiograma permite avaliar diferentes parâmetros da função ventricular direita, tais como excursão sistólica do plano do anel tricúspide ou variação fracional da área. Não existe sinal patognomônico de HAP induzida por esquistossomose, de modo que o diagnóstico diferencial deve incluir todas as outras causas de hipertensão pulmonar.^5^

Em pacientes com miocardite aguda, o ECG mostra principalmente distúrbios de repolarização.^30^ Em um estudo com 1500 soldados americanos que contraíram esquistossomose aguda durante a segunda guerra mundial, anomalias de onda T (99%) e segmentos ST (52%) foram as anomalias mais comuns. Contudo, essas mudanças foram atribuídas a efeitos colaterais de medicamentos usados no tratamento à esquistossomose utilizados na ocasião. Alterações no ECG incluindo elevação no segmento ST e depressão do segmento PR, foram apresentadas na fase aguda de até 60% dos casos.^31,32^

O ecocardiograma é o método de imagem de escolha para a avaliação da função cardíaca. Em pacientes com miocardite, o exame pode mostrar disfunção sistólica do ventrículo esquerdo com pressão alta de enchimento.^33^ Efusão pericárdica pode estar presente em até 60% dos casos com pericardite, e anormalidades na motilidade da parede em repouso podem estar presentes na isquemia miocárdica.^31,34^A ressonância magnética cardíaca (RMC) é o padrão outro para avaliação do volume e da função ventricular, e permite uma caracterização tecidual diferenciada. Assim, a RMC deve ser considerada como uma ferramenta útil em pacientes com lesão miocárdica ou envolvimento do pericárdio.^35^

**Figura 4:**
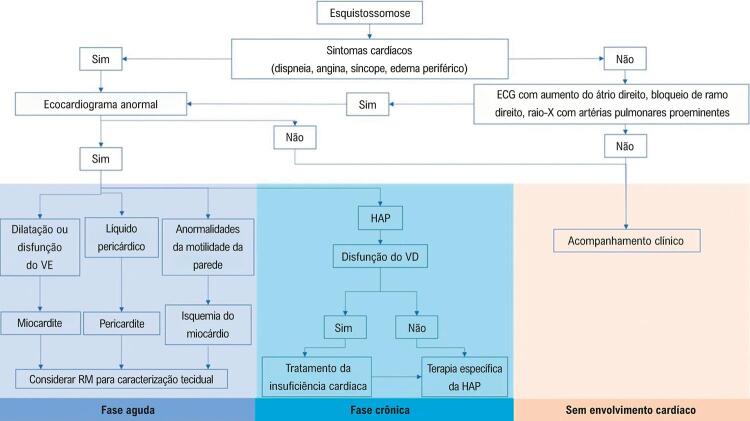
– *Algoritmo de diagnóstico proposto para o diagnóstico de envolvimento cardíaco na esquistossomose; ECG: eletrocardiograma; VE: ventrículo esquerdo; RM: ressonância magnética; HAP: hipertensão arterial pulmonar; VD: ventrículo direito.*

Um algoritmo diagnóstico para a detecção precoce do envolvimento cardiovascular como uma complicação da esquistossomose pode ser visto na Figura 4.

### Tratamento

A Tabela 2 descreve os principais agentes farmacológicos usados no manejo da esquistossomose. O medicamento de escolha para a doença é o PZQ, e derivado da pirazinoisoquinolona, com boa eficácia contra todas as espécies de Schistosoma patogênico ao homem, e taxa de cura de 80%.^36^ A principal desvantagem é o fato de que o PZQ não pode ser usado na quimioprofilaxia, uma vez que a droga é eficaz somente contra larvas maduras.^17,37^ Deve-se salientar que, quando prescrito durante a fase aguda da doença, o PZQ não previne o avanço para a fase crônica. O medicamento ainda está associado com exacerbação de sinais e sintomas em aproximadamente 50% dos casos, induzindo um tipo de resposta alérgica à destruição causada pelo parasita. Em alguns casos, essa exacerbação de sintomas pode ser fatal, causando encefalite, miocardite, e eventos pulmonares secundários à vasculite.^17,38^ O tratamento principal durante a fase aguda da doença baseia-se em corticosteroides, que atenuam a toxicidade cardíaca dos eosinófilos, complexos imunes, reações de hipersensibilidade a toxinas dos parasitas, e antígenos de superfície. Na suspeita de miocardite, o uso de PZQ deve ser adiado até recuperação cardíaca, e o manejo irá variar de acordo com dados clínicos e hemodinâmicos do paciente.^17^ Em dois casos de miocardite secundária à esquistossomose, inibidores de enzima conversora de angiotensina (IECA), e betabloqueadores foram efetivos na recuperação cardíaca.^2^ Nos pacientes cuja condição piora apesar do manejo clínico adequado, podem ser necessários suporte circulatório mecânico, dispositivo de assistência ventricular, ou oxigenação por membrana extracorpórea como ponte para o transplante ou recuperação. Ainda, em casos de doenças do pericárdio, o tratamento deve consistir em terapia com esteroides para suprimir a inflamação secundária à infecção.^3^

**Tabela 2:** – Tratamento da esquistossomose

Medicamento	Dose	Considerações especiais	Fase da doença
Corticosteroides* (prednisona)	Adulto: 1,5-2,0 mg/kg por dia por três semanas Crianças: 0,05-2,0 mg/kg por dia, três doses por dia vira oral	Reduz em 50% os níveis plasmáticos de PZQ Elimina infecção bacteriana e Strongyloides	Uso durante os dois primeiros meses após o contato com água
Praziquantel	*S haematobium, S mansoni*, 40 mg/kg por dia, uma ou duas doses por dia via oral; *S japonicum*, 60 mg/kg por dia, duas ou três doses por dia via oral	Requer uma resposta específica do hospedeiro efetiva contra o Schistosoma. Cuidado na realização de tarefas que requerem alerta durante os dois primeiros dois dias de tratamento	Ao longo do curso da doença
Oxamniquina	Contra *S mansoni*, 20 mg/kg por dia por dois ou três dias via oral	Efetiva contra cercaria invasiva, esquistossômulo maduro, te larvas maduras	Fase precoce da doença
Artemether	*S haematobium, S mansoni, S japonicum*, profilaxia: 6 mg/kg cada 2-4 semanas por mês	Efetiva contra cercaria invasiva, esquistossômulo maduro, te larvas maduras	Pode ser usada como um quimioprofilático em áreas endêmicas para indivíduos com risco elevado de infecção
**Tratamento de hipertensão arterial pulmonar associada à esquistossomose**			
Inibidores da fosfodiesterase tipo 5		Sildenafila, tadalafila, vardenafila	
antagonistas do receptor da endotelina-1		Ambrisentana, bosentana, macitentan	

* Tratamento associado para prevenir ou tratar complicações agudas

A quimioterapia no tratamento da esquistossomose tem taxa de cura entre 40% e 80%, e depende do agente quimioterápico usado, espécies de parasita, e estado nutricional do hospedeiro.^17,39^

Dados sobre a eficácia do tratamento da HAP na esquistossomose são escassos. Estudos experimentais mostraram que a terapia contra a esquistossomose reduz o remodelamento vascular pulmonar e, consequentemente, hipertensão pulmonar. No entanto. a terapia pode não ser benéfica na hipertensão pulmonar crônica, onde estudos sugerem que o remodelamento pulmonar e a HAP podem persistir mesmo após a desparasitação e desaparecimento dos ovos.^5^

Em uma pequena coorte de 12 pacientes com HAP secundária à esquistossomose, foi demonstrada melhora na classe funcional, no débito cardíaco, e na distância no teste de caminhada de seis minutos com o uso de inibidores da fosfodiesterase tipo 5 ou antagonistas do receptor da endotelina-1.^40^ Por outro lado, o manejo cirúrgico das varizes esofágicas, como a anastomose portossistêmica intra-hepática transjugular, pode aumentar a carga sobre o ventrículo direito, aumentando o risco de desvio de ovos do sistema portal.^5^

Apesar das similaridades com HAP idiopática, estudos indicam que pacientes com HAP secundária à esquistossomose apresentam um perfil hemodinâmico menos grave e taxas de sobrevida significativamente maiores.^41-43^

## Discussão

A esquistossomose é uma das DTNs mais prevalentes, que impacta de maneira desproporcional indivíduos marginalizados em regiões endêmicas. Prevalência aumentada da doença tem sido atribuída, em parte, ao aumento do turismo e visitas às regiões afetadas. A esquistossomose é uma questão de saúde púbica global, que requer melhoria em sua detecção e manejo.

O comprometimento cardiovascular da esquistossomose depende da fase da doença. Foi relatado que, na esquistossomose aguda, o paciente pode apresentar miocardite, pericardite, ou isquemia do miocárdio silenciosa, acompanhando uma reação clássica de hipersensibilidade. A HAP é a complicação mais importante da esquistossomose crônica, e um dado interessante é que os achados histopatológicos relatados na vasculatura pulmonar na HAP relacionada à esquistossomose são similares à HAP idiopática. Contudo, uma meta-análise recente mostrou um perfil hemodinâmico e uma taxa de sobrevida de cinco anos significativamente melhores em pacientes com HAP relacionada à esquistossomose em comparação à HAP idiopática.^41,43^

Existe uma lacuna considerável em termos de diagnóstico e tratamento da esquistossomose, uma vez que não existe um método considerado padrão ouro.^12,24^ História de residência ou viagem a uma área endêmica deveria levantar suspeita clínica.

Na doença aguda, o diagnóstico de um comprometimento cardíaco é um desafio, dadad sua apresentação heterogênea. A miocardite pode estar presente sem dor torácica, e somente com anormalidades de repolarização inespecíficas no ECG e altos níveis de troponina. Pericardite e isquemia miocárdica podem ser completamente assintomáticas e detectadas somente por achados eletrocardiográficos anormais. No algoritmo de diagnóstico, o ecocardiograma é sugerido como uma ferramenta chave na evolução desses pacientes. A RMC é proposta como uma ferramenta complementar, uma vez que o método possibilita a caracterização tecidual, e é preciso para a quantificação da função ventricular (Figura 4). Porém, a RMC pode não ser disponível em áreas endêmicas onde os recursos de saúde são limitados.

Em todos os pacientes com HAP, a exposição prévia deve ser investigada, e o exame microscópico de ovos na urina e fezes, teste sorológico, ou PCR devem ser realizados para estabelecer o diagnóstico dos pacientes com risco de esquistossomose.^44^ Um ecocardiograma para o diagnóstico e acompanhamento desses pacientes é essencial.

O tratamento depende do comprometimento cardíaco e a fase da doença do paciente. O envolvimento cardíaco na fase aguda deve ser tratado com corticosteroides para atenuar a resposta inflamatória. O uso de medicamentos cardíacos, tais como IECA e betabloqueadores, é empírico. Há ausência de evidência científica que oriente um tratamento definitivo desses pacientes. Na HAP, não existe um tratamento específico para a esquistossomose. Hoje, as opções limitam-se para os medicamentos para HAP idiopática.

## Conclusões

As complicações da esquistossomose impactam predominantemente indivíduos com acesso limitado à saúde. O tratamento é variável, e depende do comprometimento cardíaco, espécie de Schistosoma, e fase da doença. A maneira mais efetiva de se reduzir o impacto global da doença é pela prevenção, com foco na identificação de grupos em risco, melhoria do acesso à água potável, e do saneamento em áreas endêmicas.
