# Supplementary material for: Esquistossomose e o Coração - Em Nome das Doenças Tropicais Negligenciadas e Outras Doenças Infecciosas que Afetam o Coração (Projeto NET-Heart)
Source: Arq Bras Cardiol. 2022 Jan 11;118(5):885–93. [Article in Portuguese] doi: 10.36660/abc.20201384 (PMC9368866; doi:10.36660/abc.20201384)
Supplement: Translation

# Translation: Schistosomiasis & Heart - On Behalf of the Neglected Tropical Diseases and other Infectious Diseases affecting the Heart (the NET-Heart Project)

**Authors:** Edith Liliana Posada-Martínez, Luis Gerardo Gonzalez-Barrera, Kiera Liblik, Juan Esteban Gomez-Mesa, Clara Saldarriaga, Juan Maria Farina, Josefina Parodi, Zier Zhou, Manuel Martinez-Selles, Adrian Baranchuk

**Affiliations:** 1 Mexico Ignacio Chavez National Institute for Cardiology – Echocardiography, Juan Badiano – Mexico; 2 Mexico Medical Society of 20 of November National Medical Center – Cardiology, Mexico City – Mexico; 3 Canada Queen’s University, Kingston, Ontario – Canada; 4 Colombia Valle del Lili Foundation Internal Medicine Department, Valle del Cauca – Colombia; 5 Colombia Cardiovascular Clinic Santa Maria - Cardiology and Heart Failure, Medellin – Colombia; 6 Colombia University of Antioquia, Medellin – Colombia; 7 Argentina Clinica Olivos – Cardiology, Buenos Aires – Argentina; 8 Argentina Cardiovascular Institute of Buenos Aires, Buenos Aires – Argentina; 9 Spain Gregorio Maranon General University Hospital Cardiology Service, Madrid – Spain

**Keywords:** Schistosomiasis, Cardiovascular Diseases, Tropical Medicine

## Abstract

**Background:**

Schistosomiasis is a Neglected Tropical Disease which may lead to cardiovascular (CV) complications. However, the CV involvement in schistosomiasis has yet to be fully elucidated due to the limited number of cases and lack of reliable evidence, as schistosomiasis typically occurs in locations without adequate infrastructure for robust data collection.

**Objective:**

This systematic review aims to assess cardiovascular implications of schistosomiasis, including in the diagnosis and treatment, and propose an algorithm for screening of CV manifestations.

**Methods:**

A systematic review was performed in the MEDLINE/PubMed and LILACS databases of articles on the CV involvement in schistosomiasis.

**Results:**

Thirty-three records were considered for this review: six review articles, one systematic review, one clinical trial, 14 observational studies, seven case reports, and four cases series. CV involvement includes a wide spectrum of clinical conditions, such as myocardial ischemia, ventricular dysfunction, myocarditis, pulmonary arterial hypertension, and pericarditis.

**Conclusions:**

Cardiac complications of schistosomiasis may cause long-term disability and death. Clinical monitoring, physical examination, early electrocardiogram, and echocardiogram should be considered as key measures to detect CV involvement. Due to the lack of effective treatment of complications, sanitation and education in endemic areas are necessary for the elimination of this global health problem.

## Introduction

Schistosomiasis is a Neglected Tropical Disease (NTD) caused by blood flukes, which are trematode worms of the genus *Schistosoma*. It is endemic to rural regions with weak health infrastructure and limited access to potable water or water sanitation methods. Accordingly, schistosomiasis was added to the World Health Organization´s (WHO) 2008-2015 Global Plan to Combat NTD.^1^

According to the WHO, approximately 240 million people are affected by schistosomiasis globally, with more than 90% of cases occurring in Africa.^1^ Schistosomiasis is transmitted through penetration of Schistosoma cercariae, which are found in fresh water, into the skin. Once the larvae penetrate the skin, they invade the venous system and spread to organs such as the heart, lungs, liver, and intestines. *Schistosoma mansoni* is the main specie that infects humans and may lead to potentially life-threatening cardiovascular (CV) events. In published case reports, myocarditis, pericarditis, and myocardial ischemia have been documented in the acute phase of the disease.^2,3^ These CV outcomes are poorly understood due to a limited number of cases and lack of reporting.^4^ Patients with acute CV complications may also be asymptomatic at presentation, contributing to deficiencies in data collection. The most relevant CV complication of schistosomiasis is pulmonary arterial hypertension (PAH).^5-7^ Signs and symptoms of patients with schistosomiasis-associated PAH do not differ from those documented from other etiologies.

Schistosomiasis is estimated to be the main cause of PAH in endemic countries. Despite this, diagnosis is limited to regions with access to adequate medical equipment, and there are currently no specific medications for PAH associated with schistosomiasis. The most used pharmacologic agent is praziquantel (PZQ), which presumably prevents further progression of the disease by reversing vascular remodeling.

This systematic review is part of the “NET-Heart Project” (Neglected Tropical Diseases and Other Infectious Diseases Affecting the Heart), an initiative of the “Emerging Leaders” section of the Interamerican Society of Cardiology (IASC).^8-10^ The purpose of this study was to expand the knowledge on the impact of NTD on CV health. The aim of this review was to provide an overview of the CV involvement in schistosomiasis and to propose an algorithm for diagnosis.

## Methods

A systematic review of the literature was conducted following the design of the NET-Heart Project.^8,11^ MEDLINE/PubMed and LILACS were searched using any association of schistosomiasis with CV involvement, with no date restrictions. Only human studies, available in English were used. Papers were excluded if the full text was not available. The keywords used according to the MESH terminology were: “schistosomiasis”, “heart”, “cardiac”, “pericardium”, “pericarditis”, and “cardiovascular disease”. Articles were screened by two independent investigators (ELPM and LGGB). Interobserver agreement, assessed by Kappa statistics, was 0.93. Discrepancies were solved by consensus. An additional search was manually conducted from the reference lists of the selected articles.

The search yielded 110 articles, of which 33 articles were included in this systematic review: six review articles, one systematic review, one clinical trial, 14 observational studies, seven case reports, and four cases series (Figure 1). Table 1 (supplementary material) summarizes the studies considered for this review.

**Figure 1:** – *Flowchart of PRISMA Methodology.*

**Table 1:** – Symptoms and signs of schistosomiasis-associated pulmonary arterial hypertension

Symptoms		Signs
Pulmonary Arterial Hypertension		
Shortness of breath	Hoarseness	
Weakness	Hemoptysis	
Angina	Left parasternal lift	
Syncope	Accentuated pulmonary component of the second heart sound	
Cough	Right ventricular third heart sound	
Nausea and vomiting	Parasternal systolic murmur of tricuspid regurgitation	
	Diastolic murmur of pulmonary regurgitation	
**Right Heart Failure**		
Dyspnea		Jugular plethora
Abdominal pain		Ascites
Limb edema		Hepatomegaly
Fatigue		Peripheral edema

*Data adapted from Galie et al. 2015 ESC/ERS Guidelines for the diagnosis and treatment of pulmonary hypertension: The Joint Task Force for the Diagnosis and Treatment of Pulmonary Hypertension of the European Society of Cardiology (ESC) and the European Respiratory Society (ERS). Eur Heart J. 2016;37(1):67-119.*

## Results

### Epidemiology

Schistosomiasis is a chronic parasitic disease caused by blood flukes of the genus Schistosoma. Among those infected with schistosomes, approximately 120 million are symptomatic and 20 million have severe forms of the disease including the hepatosplenic and urinary forms.^12^

Schistosomiasis is considered endemic to South America, the Caribbean, Southeast Asia, and Africa (Figure 2). Africa is the most affected area, with more than 90% of the 41,000 deaths and 1.7 million disability-adjusted life years attributed to this disease annually. Moreover, the growth of international tourism to endemic countries has resulted in increasing number of infections in travelers. In terms of CV involvement, schistosomiasis is one of the most common causes of PAH worldwide, accounting for 30.8% of all cases of PAH in endemic areas.^13^

**Figure 2:** – *Geographical distribution of Schistosoma species; image adapted from World Health Organization^1^*

### Pathophysiology and Cardiovascular Involvement

There are five major *Schistosoma* species which infect humans. *S. mansoni, S. haematobium,* and *S. japonicum* cause most human infections.^13^ The *life cycle of Schistosoma* is shown in Figure 3.

**Figure 3:** – *Life cycle of Schistosoma.*

PAH due to schistosomiasis is particularly associated with the hepatosplenic form of *S. mansoni* infection.^14^ Schistosomiasis eggs may bypass the liver through portosystemic collateral vessels and be deposited into the lungs. The eggs cause T-helper type-2 cell-predominant immune response resulting in granuloma formation. It has been demonstrated that interleukins (IL-4 and IL-3) stimulate the release of transforming growth factor-β leading to remodeling and angiomatous and plexiform lesions.^13^Alternatively, species whose eggs are in vesical plexus can reach the lungs directly.^15-17^

The pathophysiology of schistosomiasis-associated PAH can be summarized as the following: mechanical obstruction of the pulmonary circulation by worm eggs; inflammation leading to endothelial cell dysfunction; and portal hypertension due to liver periportal fibrosis leading to pulmonary overflow and then endothelial cell dysfunction.^5^

Acute schistosomiasis, known as Katayama fever, may have CV effects, causing myocarditis, asymptomatic myocardial ischemia, and pericarditis.^2,18^ The species identified to have cardiac involvement in the acute phase are *S. haematobium, S. mansoni,* and *S. japonjicum*. Myocarditis and pericarditis during acute schistosomiasis may be related to an allergic response induced by Schistosoma, where eosinophils play an essential role.^19-21^ The mechanism of myocardial ischemia as a consequence of schistosomiasis has not been described, and is rarely reported.^2,3^ It may occur secondary to compression of the left main coronary artery due to pulmonary artery dilatation. Severe dilatation of the pulmonary artery can also result in rupture, causing cardiac tamponade.^17^

### Symptoms

PAH induced by schistosomiasis may be asymptomatic. However, in later stages of the disease, patients can present symptoms of right heart failure such as shortness of breath, bilateral edema in the lower extremities, and tachycardia. Signs and symptoms of PAH in schistosomiasis are described in Table 1.

Symptoms are non-specific, and mainly associated to right ventricular dysfunction. At onset, patients may report that their symptoms are exercise-induced. As the disease progresses, patients may develop advanced right heart failure with symptoms of systemic venous congestion.^17,22^ A hoarse voice has been noted, which is caused by compression of the recurrent laryngeal nerve. Angina has been reported in cases that have progressed to myocardial ischemia.^17^ Other clinical signs of schistosomiasis-associated PAH include hepatomegaly, ascites, peripheral edema, and elevated jugular venous pressure.^17^

In acute schistosomiasis involving the heart, patients with myocarditis may exhibit chest pain. These patients may be asymptomatic, with diagnosis made based on laboratory tests. Also, there is a report of a patient who had delayed myocardial perfusion of the septum with subendocardial enhancement two months after the acute phase, without clinical signs of ischemia.^2^

### Diagnostic Tests

The diagnosis of schistosomiasis requires an accurate and guided anamnesis, physical examination, laboratory tests, and imaging studies. In endemic areas, schistosomiasis must be highly suspected in patients with manifestations of PAH. In non-endemic countries, a recent trip to endemic areas should be considered if CV symptoms occur.

The identification of the parasite is an important part of diagnosis; however, the microscopic examination of eggs in urine (*S. haematobium*) or faeces (*S. japonicum, S. Mansoni*) is not always possible if the parasite is in the prepatent period.^23^ Additionally, available serological tests are limited, as they do not discriminate between active infection and past exposure.^12,24^ Finally, PCR-based assays have been developed for the detection of Schistosome DNA in feces, sera, and plasma during all phases of the disease.^25^

Regarding cardiac involvement, patients with PAH may show right atrial enlargement, right ventricular hypertrophy, and right bundle branch block on electrocardiogram (ECG).^19,26^ Additionally, X-ray typically shows prominent left and right pulmonary arteries. The echocardiogram is a key tool in the evaluation of these patients.^27^

In acute schistosomiasis, the echocardiogram helps to identify myocarditis, pericarditis, or myocardial ischemia.^2,28,29^ In patients with PAH, it may reveal right ventricular enlargement with septal bowing, tricuspid regurgitation, hypertrophy of the right ventricular free wall, and an increased right ventricular pressure. In addition, the echocardiogram allows to evaluate different parameters of the right ventricular function, such tricuspid annular plane systolic excursion or fractional area change. There is no pathognomonic sign of schistosomiasis-induced PAH, so the differential diagnosis should include all other causes of pulmonary hypertension.^5^

In patients with acute myocarditis, the ECG show mainly repolarization abnormalities.^30^ In a study with 1,500 American soldiers who contracted acute schistosomiasis during World War II, the anomalies of T-waves (99%) and ST segments (52%) were the most common abnormalities. However, these changes were attributed to the side-effects of anti-schistosomiasis drugs used at that moment. ECG changes including widespread ST elevation and PR depression has been demonstrated in the acute phase of up to 60% of cases.^31,32^

The echocardiogram is the first line image method for the evaluation of the cardiac function. In patients with myocarditis, it could show systolic dysfunction of the left ventricle with high filling pressures.^33^ Pericardial effusion can be present in up to 60% cases with pericarditis and wall motion abnormalities at rest may be present in myocardial ischemia.^31,34^ Cardiac magnetic resonance (CMR) is the gold standard for the evaluation of ventricular function and volume, and allows a unique tissue characterization; therefore, CMR should be consider as an useful tool in patients with myocardial injury or pericardium involvement.^35^

A diagnostic algorithm for early detection of cardiovascular involvement as a complication of schistosomiasis can be seen in Figure 4.

**Figure 4:** – *Diagnostic algorithm proposed for diagnosis of cardiac involvement in Schistosomiasis; LV: left ventricular; MRI: magnetic resonance imaging; ECG: electrocardiogram; RBBB: right bundle branch block; PAH: pulmonary arterial hypertension; RV: right ventricular.*

### Treatment

Table 2 describes the principal agents used in the management of schistosomiasis. The drug of choice for schistosomiasis is PZQ, a derivative of pyrazinoisoquinoline, with good efficacy against all species of human pathogenic schistosomes and a cure rate of 80%.^36^The main drawback is that PZQ cannot be used for chemoprophylaxis as it is only effective against mature worms.^17,37^ It should be noted that when prescribed during the acute phase of the disease, PZQ generally does not prevent the advancement to chronic phase. It is also associated with exacerbation of signs and symptoms in approximately 50% of cases, by inducing a type of allergic response to parasitic destruction. In some cases, this exacerbation of symptoms can be life-threatening causing encephalitis, myocarditis, and pulmonary events secondary to vasculitis.^17,38^

**Table 2:** – Treatment for schistosomiasis

Specific treatment of Schistosomiasis			
Drug	Dose	Special considerations	Phase of the disease
Corticosteroids* (prednisone)	Adult: 1.5-2.0 mg/kg per day by mouth for 3 weeks. Pediatric: 0.05-2.0 mg/kg per day, three doses a day by mouth	Decreases plasma levels of PZQ by 50%. Ruling out bacterial infection and strongyloidiaisis	Use within the first two months after contact with water.
Praziquantel	*S haematobium, S mansoni*, 40 mg/kg per day, one or two doses a day by mouth; *S japonicum*, 60 mg/kg per day, two or three doses a day by mouth.	Requires an effective host-specific response against the schistosome. Caution when performing tasks that require alertness on the first two days of treatment	Throughout the course of the disease
Oxamniquine	*S mansoni* only, 20 mg/kg per day, for two to three days by mouth.	It is effective against schistosomula and prevents the chronic phase	Early phase of the disease
Artemether	*S haematobium, S mansoni, S japonicum*, prophylaxis: 6 mg/kg every 2-4 weeks by mouth.	It is effective against invasive cercariae, mature schistosomula, and mature adult worms	It can be used as a chemoprophylactic in endemic areas for people at high risk of infection
**Treatment of Schistosomiasis-associated pulmonary arterial hypertension**			
Phosphodiesterase-5 inhibitors		Sildenafil, tadalafil, vardenafil	
Endothelin receptor 1 antagonists		Ambrisentan, bosentan, macitentan	

* Associated treatment to avoid or treat acute complications.

The mainstay treatment during the acute phase of the disease is based on corticosteroids, which attenuate the cardiac toxicity of eosinophils, immune complexes, hypersensitivity reactions to parasite toxins, and surface antigens. If myocarditis is suspected, the use of PZQ should be postponed until cardiac recovery and management will vary according to patient’s clinical and hemodynamic data.^17^ In two cases of myocarditis secondary to schistosomiasis, angiotensin-converting enzyme (ACE) inhibitors and beta-blockers were effective for cardiac recovery.^2^ In patients whose condition deteriorates despite optimal medical management, mechanical circulatory support, ventricular assist devices, or extracorporeal membrane oxygenation as a bridge to transplantation or recovery may be required. Similarly, in cases of pericardial disease, treatment should consist of steroidal therapy to suppress inflammation secondary to the infection.^3^

Antischistosomal chemotherapy has cure rates ranging from 40 to 80% and is especially dependent on the specific chemotherapeutic agent, species of parasite, and nutritional status of the host.^17,39^

Data on the efficacy of PAH treatment in schistosomiasis are scarce. Experimental studies showed that antischistosomal therapy reduces pulmonary vascular remodeling and, consequently, pulmonary hypertension. However, it may not be beneficial in chronic pulmonary hypertension, where studies suggest that pulmonary remodeling and PAH may persist even after complete deworming and disappearance of the eggs.^5^

In a small cohort of 12 patients with PAH secondary to schistosomiasis, improvements in functional class, cardiac output, and distance in the six-minute walk test were demonstrated using phosphodiesterase-5 inhibitors or endothelin receptor 1 antagonists.^40^ On the other hand, surgical management of oesophageal varices, like the transjugular intrahepatic portosystemic shunt, can increase the load on the right ventricle, increasing the risk of more shunting of eggs from the portal system.^5^

Despite the similarities with idiopathic PAH, studies support that patients with PAH secondary to schistosomiasis have a less severe hemodynamic profile and significantly better survival rates.^41-43^

## Discussion

Schistosomiasis is one of the most prevalent NTD, that disproportionately impacts marginalized individuals in endemic regions. Increased prevalence has been partially attributed to increase in tourism and visits to the affected regions. Schistosomiasis is a global public health issue, that requires improved detection and management.

CV involvement of schistosomiasis depends on the phase of the disease. It has been reported that in acute schistosomiasis, patient may present myocarditis, pericarditis, or silent myocardial ischemia accompanying a classic hypersensitivity reaction. PAH is the most relevant complication of chronic schistosomiasis and, interestingly, the histopathological findings reported in the pulmonary vasculature in schistosomiasis-related PAH are similar to idiopathic PAH. However, a recent meta-analysis showed significantly better hemodynamic profile and five-year survival rates in schistosomiasis-related PAH patients compared to idiopathic PAH.^41,43^

There is a considerable gap in terms of diagnosis and treatment of schistosomiasis as there is no gold standard test.^12,24^ History of living in or having traveled to an endemic area should trigger a clinical suspicion.

In the acute scenario, diagnosing cardiac involvement is a challenge due to its heterogenous presentation. Myocarditis can be present without chest pain and only with unspecific repolarization abnormalities in the ECG and high troponin levels. Similarly, pericarditis and myocardial ischemia may be completely asymptomatic and detected only by abnormal ECG findings. In the diagnostic algorithm, echocardiogram is suggested as a key tool in the course of these patients. CMR is proposed as a complementary tool as it provides tissue characterization and is accurate for quantification of ventricular function (Figure 4). However, CMR maybe not available in endemic areas where the health resources are limited.

In all patients with PAH, background exposure should be investigated, and microscopic examination of eggs in urine and feces, serological test, or PCR assay should be performed to establish the diagnosis of patients at risk for schistosomiasis.^44^ An echocardiogram is essential for diagnosis and follow-up in these patients.

Treatment is dependent on CV involvement and disease phase of the patient. Cardiac involvement in acute disease should be treated with corticosteroids to attenuate inflammatory response. The use of cardiac medications, such as ACE inhibitors and beta blockers is empiric. There is a lack of scientific evidence guiding definitive treatment of these patients. In PAH, there is no specific treatment for schistosomiasis; currently the options are limited to medications for idiopathic PAH.

## Conclusions

CV complications of schistosomiasis predominantly impacts those with limited access to healthcare. Treatment is variable, and depends on CV involvement, species of Schistosoma, and disease phase. The most effective way of reducing the global impact of the disease is by prevention, focusing on identifying groups at risk, and improving access to drinking and sanitation in endemic areas.
